# *meso*-5,15-Bis[3-(iso­propyl­idenegalacto­pyran­oxy)phen­yl]-10,20-bis­(4-methyl­phen­yl)porphyrin

**DOI:** 10.1107/S2414314624010289

**Published:** 2024-10-24

**Authors:** Mickey Vinodh, Fatemeh H. Alipour, Talal F. Al-Azemi

**Affiliations:** a Department of Chemistry, Kuwait University, PO Box 5969, Safat 13060, Kuwait; Goethe-Universität Frankfurt, Germany

**Keywords:** crystal structure, glycosyl­ated porphyrin, photosensitizers

## Abstract

The crystal structure of a glycosyl­ated porphyrin (**P_Gal2**) system, C_70_H_70_N_4_O_12_, where two iso­propyl­idene protected galactose moieties are attached to the *meso* position of a substituted tetra­aryl porphyrin is reported. This structure reveals that the parent porphyrin is planar, with the galactose moieties positioned above and below the porphyrin macrocycle. This orientation likely prevents porphyrin–porphyrin H-type aggregation, potentially enhancing its efficiency as a photosensitizer in photodynamic therapy. Notable non-bonding C—H⋯O and C—H⋯π inter­actions among adjacent **P_Gal2** systems are observed in this crystal network. Additionally, the tolyl groups of each porphyrin can engage in π–π inter­actions with the delocalized π-systems of neighboring porphyrins.

## Abstract 2

The crystal structure, non-bonding inter­actions and packing features of a novel galactose conjugated porphyrin are presented and discussed.

## Graphical abstract

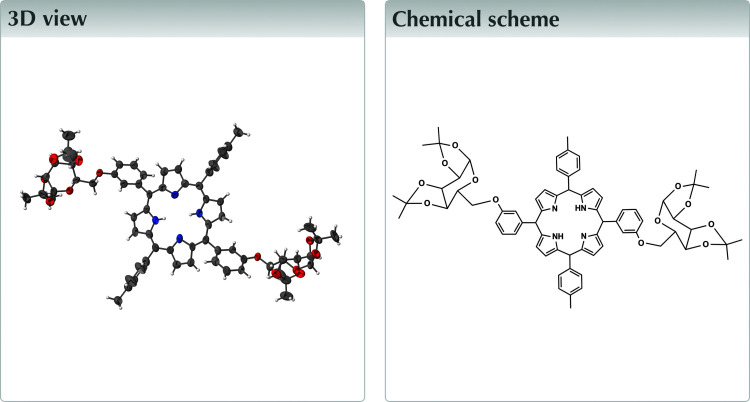

## Structure description

Porphyrins have been demonstrated to be important functional materials when bonded with other mol­ecular species (Chen *et al.*, 2021 ▸; Ciaffaglione *et al.*, 2021 ▸; Mathew *et al.*, 2021 ▸; Park *et al.*, 2021 ▸; Piradi *et al.*, 2021 ▸; Shi *et al.*, 2021 ▸; Huang *et al.*, 2022 ▸; Ishizuka *et al.*, 2022 ▸; O’Neill *et al.*, 2022 ▸; Domingo-Tafalla *et al.*, 2023 ▸; Molina *et al.*, 2023 ▸). One significant application of porphyrins and related macromolecular species is their role as photosensitizers in photodynamic therapy (PDT) for cancer treatment and other therapeutic uses (Lin *et al.*, 2020 ▸; Tian *et al.*, 2020 ▸; Zhang *et al.*, 2021 ▸; Liu *et al.*, 2023 ▸; Tian *et al.*, 2023 ▸). However, several limitations are associated with porphyrin mol­ecules when used in physiological conditions, including low solubility in bio fluids, aggregation and low tumor specificity. Intensive research is being conducted on the peripheral substitution of the porphyrin ring with suitable functional moieties to overcome these limitations. In this regard, the conjugation of carbohydrate groups to porphyrinoids has been found to be an excellent strategy to generate efficient photosensitisers for PDT (Singh *et al.*, 2015 ▸). Glyco-conjugation can improve the tumor-targeting efficiency and cellular uptake of porphyrin dyes because various types of sugar transporters, specific for different monosaccharides, are overexpressed in cancer cells. In addition to targeting tumor cells, appending biocompatible moieties to the macrocycles increases solubility in biological environments, thereby reducing aggregation and destabilizing inter­molecular inter­actions. Porphyrin aggregates are less photoactive and hence inferior in PDT (Chen *et al.*, 2004 ▸; Singh *et al.*, 2015 ▸).

In this communication, we report the crystal structure of a carbohydrate-conjugated porphyrin, where two iso­propyl­idene-protected galactose moieties are appended to a preformed porphyrin. The parent porphyrin used for the sugar conjugation is *meso*-5,15-di(3-hy­droxy­phen­yl)-10,20-di(4-tolu­yl)porphyrin. The galactose fractions are attached to the 5- and 15-positions of this porphyrin through an –O—CH_2_– spacer. The structural details and packing features of this *trans*-bis galactose porphyrin (**P_Gal2**) are presented and discussed.

The title compound crystallizes in the monoclinic crystal system, space group *P*2_1_. The ADDSYM routine implemented in *PLATON* (Spek, 2020 ▸) suggests another possible space group, namely *P*2_1_/*c*, for this crystal. However, refinement in space group *P*2_1_/*c* resulted in highly disordered galactose fractions with unacceptable *R* values. Therefore, the **P_Gal2** structure in this report was refined in space group *P*2_1._

**Figure 1:**
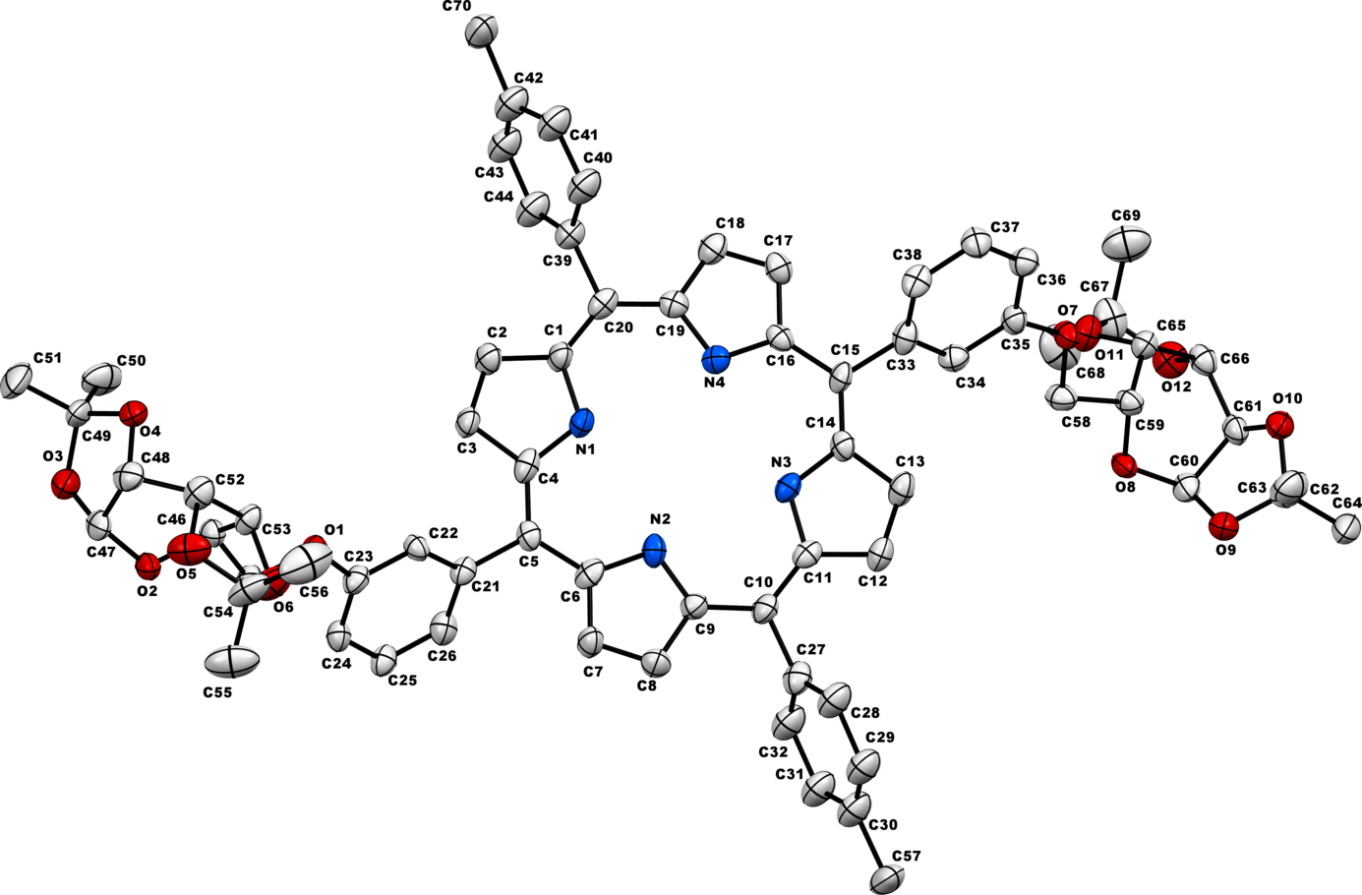
Crystal structure (displacement ellipsoid representation; 30% probability) of **P_Gal2**. Hydrogen atoms are omitted for clarity.

**Figure 2:**
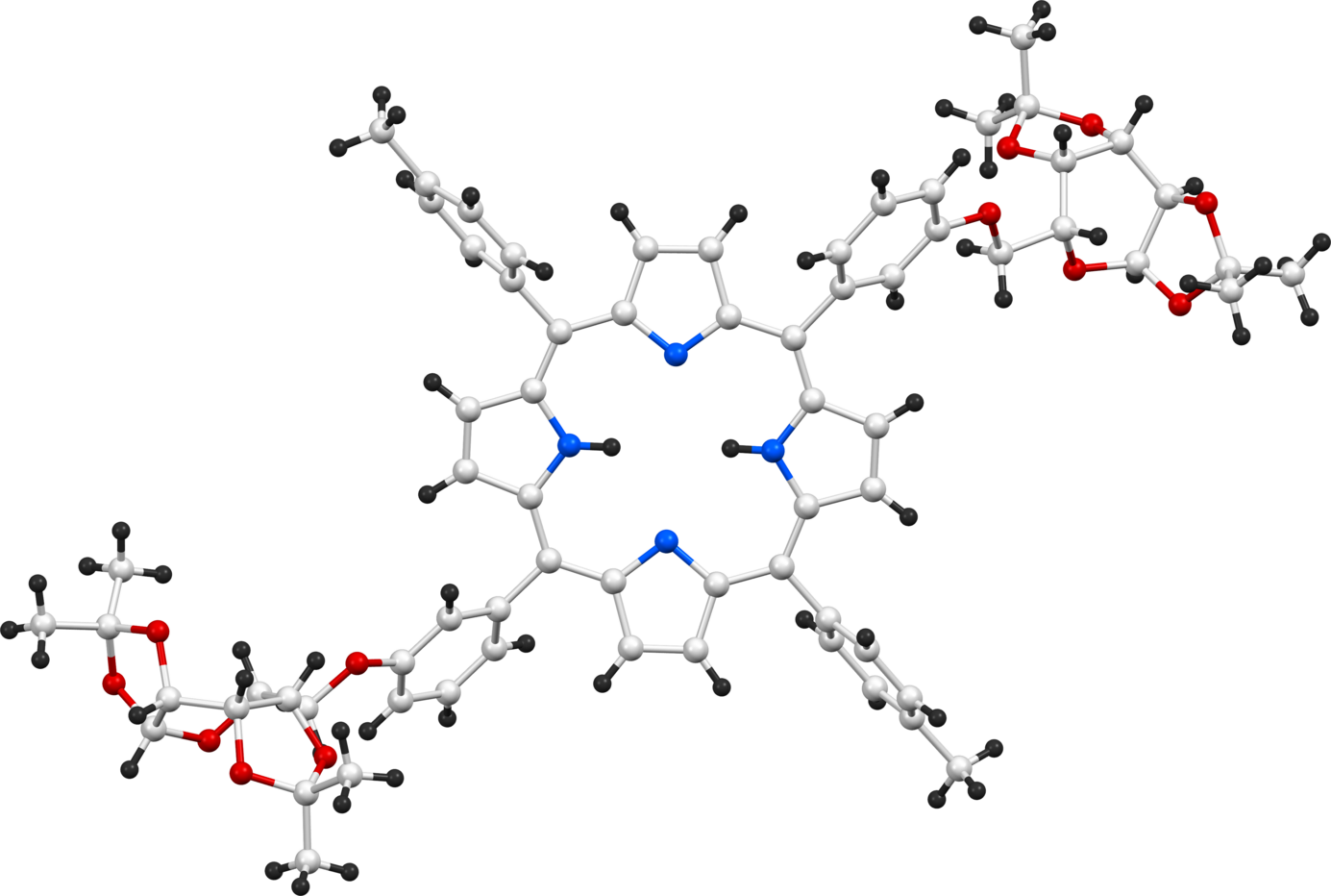
Crystal structure (ball-and-stick representation) of **P_Gal2** showing exact orientations of toluyl and galacto­pyran­ose moieties with respect to the porphyrin plane.

The structure of the porphyrin–galactose conjugate (**P_Gal2**) obtained from single-crystal diffraction analysis is depicted in Fig. 1 ▸. The porphyrin moiety is planar and the *meso*-toluyl substituents are inclined to the macrocycle by about 77° [the C1—C20—C39—C44 and C11—C10—C27—C28 torsion angles are −77.4 (14) and 76.5 (15)°, respectively] (Fig. 2 ▸). The aryl moieties linked to the sugar units are more inclined with respect to the porphyrin plane; the corresponding torsion angles are −55.8 (14)° (for C4—C5—C21—C22) and 53.8 (14)° (for C16—C15—C33—C38).

**Figure 3:**
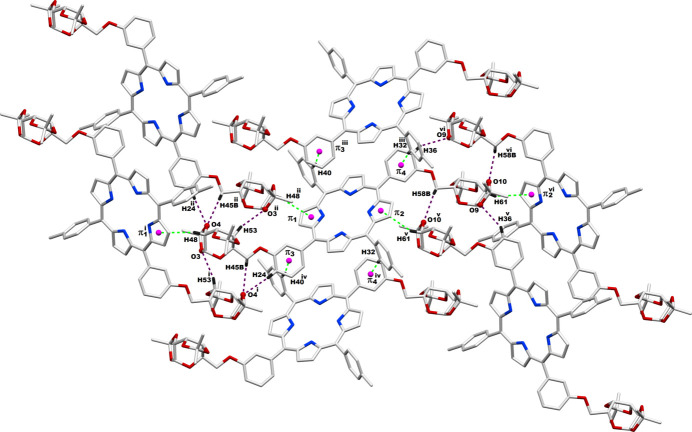
Inter­molecular inter­actions experienced by a given **P_Gal2** system with its neighboring counterparts; Symmetry codes: (i) 1 − *x*, −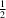 + *y*, −*z*; (ii) 1 − *x*, 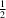 + *y*, −*z*; (iii) *x*, 1 + *y*, *z*; (iv) *x*, −1 + *y*, *z*; (v) 3 − *x*, −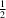 + *y*, 1 − *z*; (vi) 3 − *x*, 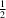 + *y*, 1 - *z.*

Due to the aryl substitution at the *meta* position, the galactose moieties are positioned such that one unit is above and the other is below the macrocyclic porphyrin plane. The –O—CH_2_– spacer provides sufficient flexibility for these sugar derivatives to comfortably locate around the chromophore. The orientation of the sugar moieties both above and below the plane of the macrocycle is sufficient to prevent H-type aggregation of the porphyrin units. There are appreciable inter­molecular C—H⋯O and C—H⋯π inter­actions between adjacent **P_Gal2** mol­ecules in the crystal network, especially in the vicinity of iso­propyl­idene-galacto­pyran­ose moieties as shown in Fig. 3 ▸. The qu­anti­tative details of these non-bonding inter­actions are given in Table 1 ▸.

**Figure 4:**
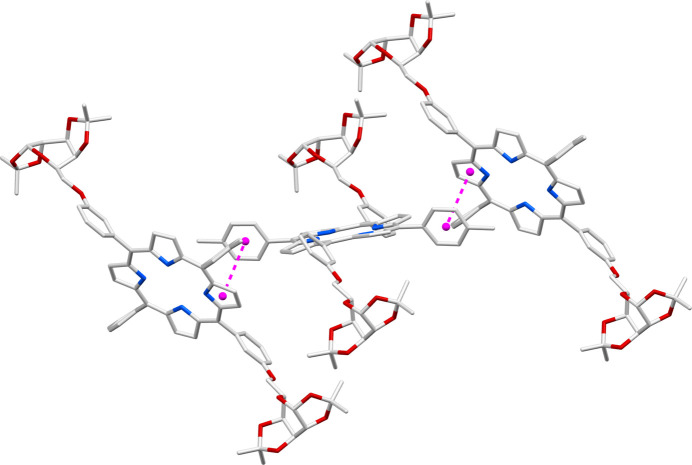
Inter­molecular π–π inter­actions between neighbouring porphyrin units in the **P_Gal2** crystal.

**Figure 5:**
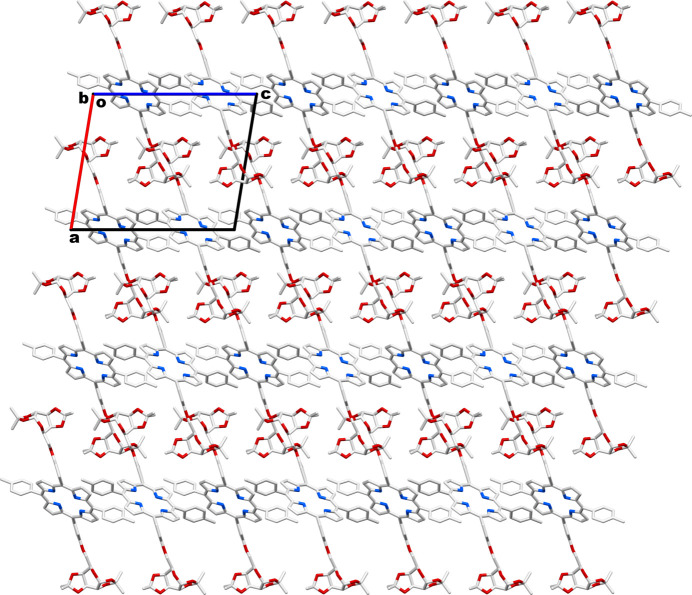
Packing pattern of **P_Gal2** systems in the crystal network.

It is also observed that the tolyl groups in the porphyrin are capable of engaging in π–π inter­actions with the pyrrole part of the delocalized porphyrin π-system, as illustrated in Fig. 4 ▸. These π–π inter­actions, along with the inter­molecular C—H⋯O and C—H⋯π inter­actions discussed above, contribute to the cohesion of the crystal. The packing pattern of this crystal (depicted in Fig. 5 ▸) is very efficient leaving no appreciable void space in the crystal network to accommodate inter­stitial solvent mol­ecules.

## Synthesis and crystallization

Tosyl­ated galacto­pyran­ose (**Gal_OTS**) was synthesized as follows. Commercially available 1,2:3,4-di-*O*-iso­propyl­idene-α-d-galacto­pyran­ose (0.52 g, 2.0 mmol) was dissolved in pyridine (20 ml) and *N,N*-di­methyl­amino­pyridine (25 mg, 5% *w*/*w*) was added. *p*-Toluene­sulfonyl­chloride (1.14 g, 6.0 mmol) was added to this mixture and stirred at room temperature for 2 h. The reaction mixture was then poured into (100 ml) of ice-cold 10% HCl solution. The precipitate formed was filtered, washed with cold water two times and dried, yielding 0.74 g (90%) of the product. ^1^H NMR (400 MHz, CDCl_3_) δ: 1.28 (*s*, 3H), 1.32 (*s*, 3H), 1.35 (*s*, 3H), 1.50 (*s*, 3H), 2.44 (*s*, 3H), 4.06 (*m*, 2H), 4.20 (*m*, 2H), 4.29 (*m*, 1H), 4.58 (*m*, 1H), 5.45 (*d*, *J* = 4.8 Hz, 1H), 7.33 (*d*, *J* = 8.4 Hz, 2H), 7.80 (*d*, *J* = 8.4 Hz, 2H). ^13^C NMR (150 MHz, CDCl_3_) δ: 21.7, 24.3, 24.9, 25.8, 25.9, 65.8, 68.2, 70.3, 70.4, 70.5, 96.1, 109.0, 109.6, 128.1, 129.8, 132.7, 144.8.

Synthesis of galactose-conjugated porphyrin (**P_Gal2**): *meso*-5,15-di(3-hy­droxy­phen­yl)-10,20-di(4-tolu­yl)porphyrin (Al-Azemi *et al.*, 2015 ▸, 168 mg, 0.25 mmol) was dissolved in DMF (25 ml) and potassium carbonate (275 mg, 2. 0 mmol) was added to this solution. The mixture was stirred at room temperature for 30 minutes. **Gal_OTS** (415 mg, 1 mmol) was then added, and the mixture was heated at 125°C for 24 h. The solvent was removed under reduced pressure, and the intended compound was purified by column chromatography using di­chloro­methane/ethyl acetate (98:2 *v*/*v*), yielding 234 mg (81%). ^1^H NMR (400 MHz, CDCl_3_) δ: −2.76 (*s*, 2H), 1.34 (*m*, 12H), 1.47 (*s*, 6H), 1.56 (*s*, 6H), 2.75 (*s*, 6H), 4.41 (*m*, 10H), 4.68 (*m*, 2H), 5.62 (*d*, *J* = 5.2 Hz, 2H), 7.41 (*m*, 2H), 7.59 (*d*, *J* = 7.6 Hz, 4H), 7.66 (*t*, *J* = 8.4 Hz, *J* = 7.6 Hz, 2H), 7.85 (*m*, 4H), 8.13 (*m*, 4H), 8.90 (*s*, 8H). ^13^C NMR (150 MHz, CDCl_3_) δ: 21.5, 24.4, 24.9, 26.0, 26.1, 66.4, 67.0, 70.6, 70.6, 71.1, 96.4, 108.8, 109.5, 114.2, 119.6, 120.2, 121.4, 127.4, 127.9, 129.8, 130.9, 134.5, 137.3, 139.2, 143.5, 157.0. MS (EI): 1158 (*M*^+^).

## Refinement

Crystal data, data collection and structure refinement details are summarized in Table 2 ▸. *DFIX* commands were applied between phenyl carbon atoms of the toluyl moieties to fix their bond length to 1.395 Å. Additionally, *SIMU* and *DELU* commands were used to restrain the thermal displacement parameters of the toluyl moieties and a few other distorted carbon/oxygen atoms in the structure.

## Supplementary Material

Crystal structure: contains datablock(s) I. DOI: 10.1107/S2414314624010289/bt4156sup1.cif

Supporting information file. DOI: 10.1107/S2414314624010289/bt4156Isup3.mol

Structure factors: contains datablock(s) I. DOI: 10.1107/S2414314624010289/bt4156Isup4.hkl

CCDC reference: 2388518

Additional supporting information:  crystallographic information; 3D view; checkCIF report

## Figures and Tables

**Table 1:** Non-bonding interactions among adjacent **P_Gal2** systems (Å, °) π1, π2, π3 and π4 are the centroids of the **[please define]** rings, respectively.

*D*—H⋯*A*	*D*—H	H⋯*A*	*D*⋯*A*	*D*—H⋯*A*
C45—H45*B*⋯O4^i^	0.99	2.55	3.18 (1)	121
C24—H24⋯O4^i^	0.95	2.60	3.54 (1)	169
C48—H48⋯π1^i^	1.00	3.18	4.120	165
C53—H53⋯O3^ii^	1.00	2.70	3.67 (1)	165
C40—H40⋯π3^iii^	0.95	2.89	3.828	171
C32—H32⋯π4^iv^	0.95	2.83	3.782	176
C58—H58*B*⋯O10^v^	0.99	2.70	3.19 (1)	110
C61—H61⋯π2^vi^	1.00	3.20	4.117	153
C36—H36⋯O9^vi^	0.95	2.70	3.56 (1)	152

Symmetry codes: (i) 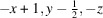; (ii) 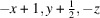; (iii) 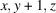; (iv) 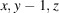; (v) 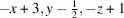; (vi) 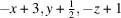.

**Table 2:** Experimental details

Crystal data	
Chemical formula	C_70_H_70_N_4_O_12_
*M*_r_	1159.30
Crystal system, space group	Monoclinic, *P*2_1_
Temperature (K)	150
*a*, *b*, *c* (Å)	16.565 (2), 9.7051 (13), 19.708 (3)
β (°)	99.376 (7)
*V* (Å^3^)	3126.0 (7)
*Z*	2
Radiation type	Mo *K*α
μ (mm^−1^)	0.08
Crystal size (mm)	0.15 × 0.07 × 0.04

Data collection	
Diffractometer	Rigaku R-AXIS RAPID
Absorption correction	Multi-scan (*ABSCOR*; Higashi, 1995 ▸)
*T*_min_, *T*_max_	0.438, 0.997
No. of measured, independent and observed [*I* > 2σ(*I*)] reflections	25359, 11348, 4159
*R*_int_	0.144
(sin θ/λ)_max_ (Å^−1^)	0.602

Refinement	
*R*[*F*^2^ > 2σ(*F*^2^)], *wR*(*F*^2^), *S*	0.082, 0.186, 0.93
No. of reflections	11348
No. of parameters	785
No. of restraints	168
H-atom treatment	H-atom parameters constrained
Δρ_max_, Δρ_min_ (e Å^−3^)	0.21, −0.20
Absolute structure	Flack *x* determined using 1142 quotients [(*I*^+^)−(*I*^−^)]/[(*I*^+^)+(*I*^−^)] (Parsons *et al.*, 2013 ▸)
Absolute structure parameter	−1.0 (10)

Computer programs: *CrystalClear* (Rigaku, 2016 ▸), *CrystalStructure* (Rigaku, 2017 ▸), *SHELXL2019/3* (Sheldrick, 2015 ▸) and *Mercury* (Macrae *et al.*, 2020 ▸).

## full crystallographic data

### meso-5,15-Bis{4-[{4,4,11,11-tetramethyl-3,5,7,10,12-pentaoxatricyclo[7.3.0.02,6]dodecan-8-yl)methoxy]phenyl}-10,20-bis(4-methylphenyl)porphyrin Crystal data

**Table:**

C_70_H_70_N_4_O_12_	*F*(000) = 1228
*M**_r_* = 1159.30	*D*_x_ = 1.232 Mg m^−^^3^
Monoclinic, *P*2_1_	Mo *K*α radiation, λ = 0.71075 Å
*a* = 16.565 (2) Å	Cell parameters from 7206 reflections
*b* = 9.7051 (13) Å	θ = 3.1–25.3°
*c* = 19.708 (3) Å	µ = 0.08 mm^−^^1^
β = 99.376 (7)°	*T* = 150 K
*V* = 3126.0 (7) Å^3^	Platelet, purple
*Z* = 2	0.15 × 0.07 × 0.04 mm

### meso-5,15-Bis{4-[{4,4,11,11-tetramethyl-3,5,7,10,12-pentaoxatricyclo[7.3.0.02,6]dodecan-8-yl)methoxy]phenyl}-10,20-bis(4-methylphenyl)porphyrin Data collection

**Table:**

Rigaku R-AXIS RAPID diffractometer	4159 reflections with *I* > 2σ(*I*)
Detector resolution: 10.000 pixels mm^-1^	*R*_int_ = 0.144
ω scans	θ_max_ = 25.3°, θ_min_ = 3.1°
Absorption correction: multi-scan (ABSCOR; Higashi, 1995)	*h* = −19→19
*T*_min_ = 0.438, *T*_max_ = 0.997	*k* = −11→11
25359 measured reflections	*l* = −23→23
11348 independent reflections	

### meso-5,15-Bis{4-[{4,4,11,11-tetramethyl-3,5,7,10,12-pentaoxatricyclo[7.3.0.02,6]dodecan-8-yl)methoxy]phenyl}-10,20-bis(4-methylphenyl)porphyrin Refinement

**Table:**

Refinement on *F*^2^	Hydrogen site location: inferred from neighbouring sites
Least-squares matrix: full	H-atom parameters constrained
*R*[*F*^2^ > 2σ(*F*^2^)] = 0.082	*w* = 1/[σ^2^(*F*_o_^2^) + (0.0622*P*)^2^] where *P* = (*F*_o_^2^ + 2*F*_c_^2^)/3
*wR*(*F*^2^) = 0.186	(Δ/σ)_max_ < 0.001
*S* = 0.93	Δρ_max_ = 0.21 e Å^−^^3^
11348 reflections	Δρ_min_ = −0.20 e Å^−^^3^
785 parameters	Absolute structure: Flack *x* determined using 1142 quotients [(*I*^+^)-(*I*^-^)]/[(*I*^+^)+(*I*^-^)] (Parsons *et al.*, 2013)
168 restraints	Absolute structure parameter: −1.0 (10)

### meso-5,15-Bis{4-[{4,4,11,11-tetramethyl-3,5,7,10,12-pentaoxatricyclo[7.3.0.02,6]dodecan-8-yl)methoxy]phenyl}-10,20-bis(4-methylphenyl)porphyrin Special details

**Table:**

Experimental. Single-crystal data were collected on Rigaku Rapid II diffractometer using Mo*K*α radiation at 150 K. The data were processed by *CrystalClear* software package (Rigaku, 2016). The structure was solved by direct methods using the *CrystalStructure* crystallographic software package (Rigaku, 2017) and the refinement was performed using *SHELXL2019*/3 (Sheldrick 2015). All non-hydrogen atoms were refined anisotropically. Hydrogen atoms are placed at calculated positions and refined using riding model with *U*_iso_(H) = 1.5Ueq(*C*-methyl) and 1.2Ueq(C) for other H atoms.
Geometry. All esds (except the esd in the dihedral angle between two l.s. planes) are estimated using the full covariance matrix. The cell esds are taken into account individually in the estimation of esds in distances, angles and torsion angles; correlations between esds in cell parameters are only used when they are defined by crystal symmetry. An approximate (isotropic) treatment of cell esds is used for estimating esds involving l.s. planes.

### meso-5,15-Bis{4-[{4,4,11,11-tetramethyl-3,5,7,10,12-pentaoxatricyclo[7.3.0.02,6]dodecan-8-yl)methoxy]phenyl}-10,20-bis(4-methylphenyl)porphyrin Fractional atomic coordinates and isotropic or equivalent isotropic displacement parameters (Å^2^)

**Table:**

	*x*	*y*	*z*	*U*_iso_*/*U*_eq_	
O1	0.6280 (4)	0.2522 (7)	0.1009 (4)	0.055 (2)	
O2	0.4526 (4)	0.0692 (7)	0.0177 (3)	0.0621 (19)	
O3	0.4074 (4)	0.0905 (8)	−0.0993 (4)	0.074 (2)	
O4	0.3810 (4)	0.3139 (7)	−0.0770 (3)	0.0605 (19)	
O5	0.3201 (5)	0.2320 (9)	0.0859 (5)	0.086 (3)	
O6	0.4501 (5)	0.2353 (9)	0.1429 (4)	0.084 (3)	
O7	1.3653 (4)	1.3074 (7)	0.3981 (4)	0.0562 (19)	
O8	1.5313 (3)	1.1102 (6)	0.4867 (3)	0.0567 (18)	
O9	1.5835 (4)	1.1426 (8)	0.6019 (3)	0.070 (2)	
O10	1.6183 (4)	1.3590 (7)	0.5719 (3)	0.060 (2)	
O11	1.5438 (5)	1.2402 (8)	0.3574 (4)	0.072 (2)	
O12	1.6742 (5)	1.2566 (8)	0.4102 (5)	0.077 (3)	
N1	0.9282 (5)	0.6798 (10)	0.1592 (4)	0.052 (2)	
H1	0.954839	0.711788	0.198271	0.062*	
N2	0.9761 (5)	0.5749 (9)	0.2972 (4)	0.053 (2)	
N3	1.0756 (5)	0.8169 (10)	0.3437 (4)	0.049 (2)	
H3	1.047742	0.788022	0.304487	0.058*	
N4	1.0283 (5)	0.9229 (9)	0.2052 (4)	0.051 (2)	
C1	0.9184 (6)	0.7499 (13)	0.0974 (6)	0.051 (3)	
C2	0.8702 (6)	0.6644 (13)	0.0490 (5)	0.058 (3)	
H2	0.851864	0.686988	0.002081	0.069*	
C3	0.8544 (6)	0.5465 (13)	0.0794 (5)	0.057 (3)	
H3A	0.824321	0.470900	0.057576	0.068*	
C4	0.8907 (6)	0.5536 (13)	0.1512 (5)	0.054 (3)	
C5	0.8838 (6)	0.4574 (12)	0.2028 (6)	0.051 (3)	
C6	0.9206 (6)	0.4714 (12)	0.2727 (6)	0.053 (3)	
C7	0.9028 (6)	0.3823 (12)	0.3261 (5)	0.060 (3)	
H7	0.865585	0.307189	0.321197	0.072*	
C8	0.9496 (7)	0.4261 (12)	0.3849 (6)	0.063 (3)	
H8	0.952624	0.386823	0.429422	0.076*	
C9	0.9933 (6)	0.5434 (12)	0.3664 (5)	0.051 (3)	
C10	1.0500 (6)	0.6170 (13)	0.4145 (5)	0.056 (3)	
C11	1.0844 (7)	0.7422 (13)	0.4040 (6)	0.054 (3)	
C12	1.1346 (6)	0.8275 (14)	0.4543 (5)	0.059 (3)	
H12	1.153168	0.803771	0.500976	0.070*	
C13	1.1507 (6)	0.9472 (14)	0.4235 (5)	0.061 (3)	
H13	1.180582	1.022649	0.445768	0.073*	
C14	1.1153 (6)	0.9417 (13)	0.3519 (6)	0.053 (3)	
C15	1.1196 (6)	1.0370 (12)	0.3012 (5)	0.050 (3)	
C16	1.0816 (7)	1.0226 (11)	0.2316 (6)	0.049 (3)	
C17	1.0976 (6)	1.1156 (12)	0.1770 (5)	0.058 (3)	
H17	1.133870	1.192042	0.181368	0.070*	
C18	1.0498 (6)	1.0703 (13)	0.1188 (6)	0.064 (3)	
H18	1.045910	1.110858	0.074498	0.077*	
C19	1.0070 (6)	0.9524 (12)	0.1354 (5)	0.052 (3)	
C20	0.9532 (6)	0.8757 (12)	0.0885 (5)	0.051 (3)	
C21	0.8339 (6)	0.3312 (13)	0.1840 (5)	0.049 (3)	
C22	0.7523 (6)	0.3413 (12)	0.1525 (5)	0.048 (3)	
H22	0.727941	0.429648	0.144192	0.057*	
C23	0.7065 (7)	0.2250 (13)	0.1333 (6)	0.053 (3)	
C24	0.7389 (6)	0.0975 (13)	0.1464 (5)	0.053 (3)	
H24	0.706349	0.017820	0.134432	0.064*	
C25	0.8204 (6)	0.0842 (12)	0.1774 (5)	0.055 (3)	
H25	0.844028	−0.004627	0.185696	0.066*	
C26	0.8656 (8)	0.1981 (14)	0.1955 (6)	0.060 (3)	
H26	0.920947	0.187429	0.216917	0.071*	
C27	1.0745 (6)	0.5497 (12)	0.4833 (5)	0.065 (3)	
C28	1.0459 (6)	0.5893 (12)	0.5418 (5)	0.067 (3)	
H28	1.007720	0.662807	0.539723	0.081*	
C29	1.0724 (7)	0.5228 (12)	0.6048 (6)	0.072 (3)	
H29	1.053241	0.554033	0.644960	0.086*	
C30	1.1252 (7)	0.4141 (13)	0.6092 (6)	0.076 (3)	
C31	1.1543 (9)	0.3733 (16)	0.5514 (6)	0.110 (4)	
H31	1.192796	0.300186	0.553893	0.132*	
C32	1.1274 (8)	0.4387 (14)	0.4884 (6)	0.099 (4)	
H32	1.146059	0.406078	0.448177	0.119*	
C33	1.1643 (6)	1.1698 (14)	0.3184 (5)	0.055 (3)	
C34	1.2460 (6)	1.1691 (13)	0.3504 (5)	0.055 (3)	
H34	1.273934	1.084639	0.361723	0.066*	
C35	1.2854 (7)	1.2934 (13)	0.3654 (6)	0.052 (3)	
C36	1.2480 (7)	1.4171 (14)	0.3472 (5)	0.058 (3)	
H36	1.277357	1.501065	0.355812	0.069*	
C37	1.1666 (7)	1.4177 (14)	0.3162 (5)	0.062 (3)	
H37	1.139301	1.502405	0.303901	0.074*	
C38	1.1250 (7)	1.2925 (13)	0.3030 (6)	0.054 (3)	
H38	1.068714	1.292745	0.283148	0.065*	
C39	0.9263 (6)	0.9432 (12)	0.0195 (5)	0.063 (3)	
C40	0.8759 (8)	1.0579 (14)	0.0147 (6)	0.098 (4)	
H40	0.859411	1.091771	0.055595	0.118*	
C41	0.8481 (9)	1.1264 (16)	−0.0468 (5)	0.110 (4)	
H41	0.814105	1.205354	−0.047514	0.132*	
C42	0.8706 (8)	1.0778 (13)	−0.1061 (5)	0.078 (3)	
C43	0.9260 (7)	0.9735 (12)	−0.1015 (5)	0.073 (3)	
H43	0.946518	0.945562	−0.141692	0.088*	
C44	0.9535 (6)	0.9070 (12)	−0.0399 (5)	0.063 (3)	
H44	0.992116	0.834364	−0.038827	0.076*	
C45	0.5781 (6)	0.1336 (11)	0.0795 (5)	0.060 (3)	
H45A	0.565493	0.082156	0.119858	0.072*	
H45B	0.606285	0.071094	0.051268	0.072*	
C46	0.5012 (6)	0.1911 (11)	0.0378 (5)	0.053 (3)	
H46	0.514950	0.237211	−0.004232	0.064*	
C47	0.3836 (6)	0.0943 (12)	−0.0331 (5)	0.065 (3)	
H47	0.341382	0.021761	−0.030353	0.079*	
C48	0.3465 (7)	0.2341 (13)	−0.0271 (7)	0.063 (4)	
H48	0.285845	0.228251	−0.041341	0.075*	
C49	0.3927 (9)	0.2233 (13)	−0.1306 (6)	0.070 (4)	
C50	0.4691 (9)	0.2674 (12)	−0.1574 (6)	0.095 (5)	
H50A	0.479188	0.204350	−0.194016	0.114*	
H50B	0.515870	0.265140	−0.119912	0.114*	
H50C	0.461893	0.361185	−0.175812	0.114*	
C51	0.3169 (9)	0.2198 (14)	−0.1862 (7)	0.113 (6)	
H51A	0.268674	0.198315	−0.165108	0.136*	
H51B	0.323673	0.148938	−0.220204	0.136*	
H51C	0.309512	0.309840	−0.208816	0.136*	
C52	0.3656 (6)	0.3051 (12)	0.0420 (5)	0.064 (3)	
H52	0.348897	0.404161	0.038176	0.077*	
C53	0.4551 (5)	0.2910 (11)	0.0764 (4)	0.054 (3)	
H53	0.482634	0.382993	0.080629	0.065*	
C54	0.3668 (10)	0.2366 (17)	0.1536 (8)	0.092 (4)	
C55	0.3511 (8)	0.1118 (16)	0.1918 (6)	0.117 (5)	
H55A	0.363465	0.030114	0.166114	0.140*	
H55B	0.293452	0.109818	0.197542	0.140*	
H55C	0.385852	0.112468	0.237035	0.140*	
C56	0.3521 (9)	0.3716 (16)	0.1898 (6)	0.125 (5)	
H56A	0.296732	0.371387	0.201381	0.150*	
H56B	0.357796	0.449309	0.159176	0.150*	
H56C	0.392188	0.380598	0.231969	0.150*	
C57	1.1547 (8)	0.3440 (15)	0.6773 (6)	0.101 (5)	
H57A	1.210454	0.374699	0.695308	0.121*	
H57B	1.154416	0.243935	0.670676	0.121*	
H57C	1.118369	0.367984	0.710108	0.121*	
C58	1.4079 (6)	1.1838 (11)	0.4224 (5)	0.060 (3)	
H58A	1.416401	1.123981	0.383511	0.072*	
H58B	1.377085	1.131685	0.452991	0.072*	
C59	1.4896 (6)	1.2342 (10)	0.4619 (6)	0.054 (3)	
H59	1.479168	1.290577	0.502015	0.065*	
C60	1.6037 (6)	1.1311 (11)	0.5346 (5)	0.059 (3)	
H60	1.641813	1.051695	0.532919	0.071*	
C61	1.6481 (7)	1.2662 (11)	0.5249 (6)	0.058 (4)	
H61	1.708568	1.253709	0.537920	0.069*	
C62	1.6012 (9)	1.2775 (13)	0.6297 (7)	0.074 (4)	
C63	1.5287 (8)	1.3348 (14)	0.6537 (5)	0.090 (4)	
H63A	1.514538	1.277231	0.690929	0.107*	
H63B	1.482699	1.336476	0.615538	0.107*	
H63C	1.540511	1.428708	0.670703	0.107*	
C64	1.6765 (9)	1.2745 (12)	0.6847 (7)	0.096 (5)	
H64A	1.692052	1.368875	0.698831	0.115*	
H64B	1.721544	1.230188	0.666383	0.115*	
H64C	1.664547	1.222335	0.724492	0.115*	
C65	1.5385 (6)	1.3168 (12)	0.4201 (5)	0.056 (3)	
H65	1.512922	1.409154	0.409102	0.068*	
C66	1.6281 (5)	1.3319 (11)	0.4543 (5)	0.057 (3)	
H66	1.644508	1.431172	0.456519	0.068*	
C67	1.6247 (9)	1.2509 (15)	0.3435 (8)	0.082 (4)	
C68	1.6470 (9)	1.1198 (14)	0.3099 (6)	0.120 (5)	
H68A	1.614297	1.111910	0.263887	0.144*	
H68B	1.636055	1.040584	0.337821	0.144*	
H68C	1.705259	1.121701	0.306012	0.144*	
C69	1.6335 (9)	1.3803 (16)	0.3025 (6)	0.110 (5)	
H69A	1.596684	1.375469	0.258318	0.132*	
H69B	1.690162	1.388572	0.294416	0.132*	
H69C	1.619521	1.460792	0.328301	0.132*	
C70	0.8407 (8)	1.1557 (16)	−0.1738 (5)	0.102 (4)	
H70A	0.796702	1.103204	−0.201547	0.122*	
H70B	0.886274	1.166548	−0.199439	0.122*	
H70C	0.820220	1.246651	−0.163348	0.122*	

### meso-5,15-Bis{4-[{4,4,11,11-tetramethyl-3,5,7,10,12-pentaoxatricyclo[7.3.0.02,6]dodecan-8-yl)methoxy]phenyl}-10,20-bis(4-methylphenyl)porphyrin Atomic displacement parameters (Å^2^)

**Table:**

	*U*^11^	*U*^22^	*U*^33^	*U*^12^	*U*^13^	*U*^23^
O1	0.039 (4)	0.055 (5)	0.065 (5)	−0.007 (4)	−0.007 (4)	0.004 (4)
O2	0.061 (4)	0.048 (5)	0.068 (4)	−0.011 (4)	−0.017 (3)	0.005 (3)
O3	0.094 (5)	0.060 (6)	0.060 (5)	0.002 (4)	−0.011 (4)	−0.001 (4)
O4	0.072 (5)	0.050 (5)	0.055 (5)	0.003 (4)	−0.005 (4)	0.000 (4)
O5	0.058 (5)	0.113 (7)	0.093 (6)	0.013 (5)	0.027 (5)	0.035 (5)
O6	0.074 (5)	0.122 (7)	0.059 (6)	0.011 (5)	0.020 (4)	0.017 (4)
O7	0.041 (4)	0.047 (4)	0.074 (5)	−0.003 (4)	−0.010 (3)	0.009 (4)
O8	0.054 (4)	0.036 (4)	0.074 (4)	−0.002 (3)	−0.009 (3)	0.005 (3)
O9	0.088 (5)	0.054 (6)	0.064 (5)	−0.008 (4)	−0.001 (4)	0.003 (4)
O10	0.065 (5)	0.051 (5)	0.061 (5)	0.004 (4)	0.003 (4)	−0.004 (4)
O11	0.060 (5)	0.090 (6)	0.064 (6)	−0.006 (4)	0.005 (4)	−0.012 (4)
O12	0.052 (5)	0.090 (7)	0.091 (7)	0.001 (4)	0.017 (5)	−0.003 (5)
N1	0.045 (5)	0.063 (7)	0.043 (6)	−0.005 (5)	−0.003 (4)	−0.007 (5)
N2	0.037 (5)	0.064 (7)	0.054 (6)	−0.002 (5)	0.000 (4)	−0.013 (5)
N3	0.041 (5)	0.061 (6)	0.043 (6)	0.003 (5)	0.001 (4)	−0.007 (5)
N4	0.041 (5)	0.054 (6)	0.058 (6)	−0.002 (5)	0.010 (4)	0.003 (5)
C1	0.038 (6)	0.071 (7)	0.042 (7)	−0.005 (5)	0.001 (5)	−0.001 (6)
C2	0.040 (6)	0.073 (9)	0.057 (7)	−0.013 (7)	0.001 (6)	0.007 (7)
C3	0.047 (7)	0.069 (9)	0.051 (7)	−0.005 (6)	−0.005 (6)	−0.011 (7)
C4	0.041 (7)	0.073 (10)	0.044 (7)	0.007 (7)	−0.003 (6)	−0.007 (6)
C5	0.032 (6)	0.057 (8)	0.062 (8)	0.000 (6)	0.003 (6)	−0.008 (7)
C6	0.026 (6)	0.079 (9)	0.051 (7)	0.002 (6)	−0.003 (5)	0.006 (7)
C7	0.054 (7)	0.069 (9)	0.055 (7)	−0.014 (6)	0.001 (6)	−0.005 (6)
C8	0.068 (8)	0.064 (9)	0.059 (7)	−0.004 (7)	0.010 (6)	0.005 (7)
C9	0.044 (7)	0.061 (9)	0.049 (7)	−0.003 (6)	0.006 (6)	0.002 (6)
C10	0.048 (6)	0.064 (9)	0.054 (7)	0.005 (6)	−0.004 (5)	0.000 (7)
C11	0.050 (8)	0.066 (8)	0.045 (8)	0.010 (6)	0.001 (6)	−0.002 (7)
C12	0.040 (6)	0.085 (10)	0.048 (7)	−0.003 (7)	−0.001 (5)	−0.018 (8)
C13	0.045 (7)	0.083 (10)	0.051 (7)	−0.015 (7)	−0.003 (6)	−0.004 (7)
C14	0.033 (6)	0.066 (9)	0.060 (8)	−0.004 (6)	0.004 (6)	−0.008 (7)
C15	0.033 (6)	0.068 (8)	0.046 (7)	−0.002 (6)	−0.005 (5)	−0.004 (6)
C16	0.043 (7)	0.047 (7)	0.058 (7)	0.002 (6)	0.013 (6)	0.002 (6)
C17	0.052 (7)	0.055 (8)	0.068 (8)	−0.003 (6)	0.008 (6)	−0.007 (7)
C18	0.052 (7)	0.074 (10)	0.062 (8)	−0.010 (7)	0.000 (6)	0.003 (7)
C19	0.049 (7)	0.053 (8)	0.053 (7)	0.006 (7)	0.007 (6)	0.001 (6)
C20	0.042 (6)	0.072 (8)	0.041 (6)	0.007 (6)	0.011 (5)	0.002 (6)
C21	0.040 (6)	0.059 (9)	0.046 (7)	0.003 (7)	0.001 (5)	−0.010 (6)
C22	0.043 (7)	0.039 (7)	0.057 (7)	0.001 (6)	−0.001 (5)	0.003 (6)
C23	0.040 (7)	0.060 (9)	0.055 (8)	0.006 (6)	−0.005 (6)	0.002 (6)
C24	0.043 (6)	0.058 (8)	0.055 (7)	−0.011 (6)	−0.004 (5)	−0.007 (6)
C25	0.056 (7)	0.052 (8)	0.053 (7)	0.014 (6)	−0.001 (6)	−0.013 (6)
C26	0.055 (8)	0.069 (9)	0.053 (8)	0.008 (8)	0.004 (6)	−0.007 (7)
C27	0.055 (6)	0.082 (7)	0.054 (5)	0.005 (5)	−0.001 (5)	−0.003 (6)
C28	0.067 (6)	0.074 (7)	0.061 (6)	−0.015 (6)	0.010 (5)	−0.002 (5)
C29	0.072 (7)	0.086 (8)	0.056 (5)	−0.027 (5)	0.009 (5)	−0.005 (6)
C30	0.074 (7)	0.094 (8)	0.055 (5)	−0.014 (6)	0.000 (5)	0.008 (6)
C31	0.117 (8)	0.137 (9)	0.075 (6)	0.050 (7)	0.007 (6)	0.023 (7)
C32	0.100 (8)	0.135 (9)	0.060 (6)	0.046 (7)	0.005 (6)	0.012 (7)
C33	0.035 (7)	0.071 (9)	0.058 (7)	−0.001 (7)	0.005 (6)	−0.006 (7)
C34	0.048 (7)	0.055 (8)	0.061 (7)	0.002 (7)	0.003 (6)	0.003 (6)
C35	0.043 (7)	0.063 (9)	0.050 (7)	−0.006 (7)	0.002 (6)	0.005 (6)
C36	0.049 (7)	0.063 (9)	0.059 (7)	0.001 (6)	0.000 (5)	0.005 (6)
C37	0.050 (7)	0.063 (9)	0.070 (8)	0.009 (6)	0.001 (6)	0.006 (7)
C38	0.040 (7)	0.068 (9)	0.055 (7)	0.007 (7)	0.007 (6)	−0.010 (7)
C39	0.057 (6)	0.083 (7)	0.048 (5)	−0.001 (5)	0.007 (5)	0.005 (6)
C40	0.108 (8)	0.136 (9)	0.050 (5)	0.042 (7)	0.009 (6)	0.014 (6)
C41	0.128 (8)	0.140 (9)	0.058 (6)	0.043 (7)	0.000 (7)	0.016 (6)
C42	0.086 (7)	0.102 (8)	0.041 (5)	−0.016 (6)	−0.003 (5)	−0.002 (5)
C43	0.074 (7)	0.102 (8)	0.045 (5)	−0.021 (6)	0.015 (5)	−0.004 (6)
C44	0.060 (6)	0.080 (7)	0.051 (5)	−0.011 (5)	0.014 (5)	0.000 (5)
C45	0.048 (6)	0.065 (8)	0.064 (6)	−0.002 (6)	0.002 (5)	−0.007 (6)
C46	0.044 (6)	0.050 (7)	0.063 (7)	−0.008 (6)	−0.001 (6)	0.010 (6)
C47	0.048 (6)	0.059 (9)	0.079 (8)	−0.011 (6)	−0.019 (6)	0.006 (7)
C48	0.041 (7)	0.067 (9)	0.075 (10)	0.001 (6)	−0.004 (7)	0.019 (7)
C49	0.102 (11)	0.063 (10)	0.040 (8)	−0.007 (8)	−0.008 (8)	0.003 (6)
C50	0.147 (13)	0.091 (11)	0.053 (8)	0.012 (9)	0.036 (8)	0.003 (6)
C51	0.144 (14)	0.100 (13)	0.071 (10)	0.005 (10)	−0.054 (10)	0.002 (8)
C52	0.047 (6)	0.083 (9)	0.062 (7)	−0.006 (6)	0.009 (5)	0.003 (7)
C53	0.036 (5)	0.082 (9)	0.045 (6)	−0.002 (6)	0.006 (5)	0.001 (6)
C54	0.081 (7)	0.122 (8)	0.079 (7)	0.015 (7)	0.028 (6)	0.029 (6)
C55	0.098 (9)	0.139 (11)	0.123 (10)	0.034 (9)	0.051 (8)	0.059 (9)
C56	0.141 (12)	0.149 (11)	0.093 (9)	0.064 (11)	0.041 (8)	0.018 (8)
C57	0.107 (10)	0.115 (11)	0.068 (7)	−0.038 (9)	−0.021 (7)	0.026 (8)
C58	0.054 (7)	0.048 (7)	0.076 (8)	−0.001 (6)	0.002 (6)	0.012 (6)
C59	0.052 (7)	0.041 (7)	0.066 (7)	−0.009 (6)	0.003 (6)	0.010 (6)
C60	0.048 (6)	0.059 (8)	0.066 (7)	0.001 (6)	−0.009 (6)	−0.002 (6)
C61	0.047 (8)	0.051 (9)	0.071 (10)	−0.005 (6)	−0.003 (7)	−0.002 (7)
C62	0.091 (11)	0.065 (11)	0.061 (9)	0.010 (8)	−0.003 (8)	0.008 (7)
C63	0.120 (11)	0.095 (10)	0.056 (7)	0.005 (10)	0.018 (7)	−0.005 (7)
C64	0.137 (13)	0.057 (10)	0.074 (10)	0.002 (8)	−0.038 (10)	−0.004 (7)
C65	0.053 (6)	0.062 (8)	0.051 (6)	−0.005 (6)	−0.001 (5)	0.002 (6)
C66	0.044 (6)	0.054 (7)	0.072 (7)	0.000 (6)	0.007 (5)	−0.002 (6)
C67	0.069 (9)	0.093 (11)	0.088 (11)	0.002 (8)	0.022 (8)	−0.018 (8)
C68	0.157 (14)	0.096 (12)	0.127 (12)	0.004 (11)	0.079 (11)	−0.037 (10)
C69	0.113 (11)	0.127 (14)	0.100 (10)	0.006 (10)	0.048 (8)	0.026 (10)
C70	0.122 (10)	0.120 (11)	0.056 (7)	−0.030 (9)	−0.008 (7)	0.021 (8)

### meso-5,15-Bis{4-[{4,4,11,11-tetramethyl-3,5,7,10,12-pentaoxatricyclo[7.3.0.02,6]dodecan-8-yl)methoxy]phenyl}-10,20-bis(4-methylphenyl)porphyrin Geometric parameters (Å, º)

**Table:**

O1—C23	1.378 (12)	C31—H31	0.9500
O1—C45	1.439 (11)	C32—H32	0.9500
O2—C47	1.412 (10)	C33—C38	1.368 (15)
O2—C46	1.450 (11)	C33—C34	1.397 (13)
O3—C47	1.424 (11)	C34—C35	1.380 (15)
O3—C49	1.432 (13)	C34—H34	0.9500
O4—C49	1.412 (14)	C35—C36	1.372 (15)
O4—C48	1.441 (13)	C36—C37	1.387 (14)
O5—C52	1.427 (12)	C36—H36	0.9500
O5—C54	1.430 (15)	C37—C38	1.401 (16)
O6—C54	1.429 (15)	C37—H37	0.9500
O6—C53	1.433 (11)	C38—H38	0.9500
O7—C35	1.380 (12)	C39—C44	1.367 (9)
O7—C58	1.435 (11)	C39—C40	1.385 (10)
O8—C60	1.416 (9)	C40—C41	1.393 (10)
O8—C59	1.433 (11)	C40—H40	0.9500
O9—C60	1.423 (11)	C41—C42	1.367 (10)
O9—C62	1.432 (14)	C41—H41	0.9500
O10—C61	1.435 (13)	C42—C43	1.359 (11)
O10—C62	1.453 (14)	C42—C70	1.544 (15)
O11—C67	1.414 (15)	C43—C44	1.386 (10)
O11—C65	1.457 (12)	C43—H43	0.9500
O12—C67	1.433 (15)	C44—H44	0.9500
O12—C66	1.444 (11)	C45—C46	1.507 (12)
N1—C4	1.370 (14)	C45—H45A	0.9900
N1—C1	1.381 (13)	C45—H45B	0.9900
N1—H1	0.8800	C46—C53	1.514 (14)
N2—C9	1.381 (11)	C46—H46	1.0000
N2—C6	1.395 (13)	C47—C48	1.503 (15)
N3—C14	1.374 (14)	C47—H47	1.0000
N3—C11	1.378 (13)	C48—C52	1.513 (15)
N3—H3	0.8800	C48—H48	1.0000
N4—C16	1.356 (13)	C49—C50	1.511 (17)
N4—C19	1.394 (12)	C49—C51	1.527 (16)
C1—C20	1.373 (14)	C50—H50A	0.9800
C1—C2	1.410 (14)	C50—H50B	0.9800
C2—C3	1.338 (14)	C50—H50C	0.9800
C2—H2	0.9500	C51—H51A	0.9800
C3—C4	1.446 (13)	C51—H51B	0.9800
C3—H3A	0.9500	C51—H51C	0.9800
C4—C5	1.399 (14)	C52—C53	1.531 (12)
C5—C6	1.419 (13)	C52—H52	1.0000
C5—C21	1.490 (15)	C53—H53	1.0000
C6—C7	1.429 (13)	C54—C55	1.470 (17)
C7—C8	1.354 (13)	C54—C56	1.530 (18)
C7—H7	0.9500	C55—H55A	0.9800
C8—C9	1.429 (14)	C55—H55B	0.9800
C8—H8	0.9500	C55—H55C	0.9800
C9—C10	1.414 (14)	C56—H56A	0.9800
C10—C11	1.373 (14)	C56—H56B	0.9800
C10—C27	1.501 (14)	C56—H56C	0.9800
C11—C12	1.446 (14)	C57—H57A	0.9800
C12—C13	1.356 (15)	C57—H57B	0.9800
C12—H12	0.9500	C57—H57C	0.9800
C13—C14	1.439 (13)	C58—C59	1.527 (12)
C13—H13	0.9500	C58—H58A	0.9900
C14—C15	1.371 (14)	C58—H58B	0.9900
C15—C16	1.421 (13)	C59—C65	1.480 (13)
C15—C33	1.498 (16)	C59—H59	1.0000
C16—C17	1.461 (13)	C60—C61	1.530 (14)
C17—C18	1.356 (13)	C60—H60	1.0000
C17—H17	0.9500	C61—C66	1.518 (14)
C18—C19	1.413 (14)	C61—H61	1.0000
C18—H18	0.9500	C62—C63	1.470 (16)
C19—C20	1.390 (14)	C62—C64	1.513 (16)
C20—C39	1.510 (13)	C63—H63A	0.9800
C21—C22	1.396 (13)	C63—H63B	0.9800
C21—C26	1.399 (16)	C63—H63C	0.9800
C22—C23	1.378 (14)	C64—H64A	0.9800
C22—H22	0.9500	C64—H64B	0.9800
C23—C24	1.357 (14)	C64—H64C	0.9800
C24—C25	1.394 (13)	C65—C66	1.534 (12)
C24—H24	0.9500	C65—H65	1.0000
C25—C26	1.350 (15)	C66—H66	1.0000
C25—H25	0.9500	C67—C68	1.508 (16)
C26—H26	0.9500	C67—C69	1.513 (17)
C27—C28	1.370 (10)	C68—H68A	0.9800
C27—C32	1.382 (10)	C68—H68B	0.9800
C28—C29	1.404 (10)	C68—H68C	0.9800
C28—H28	0.9500	C69—H69A	0.9800
C29—C30	1.363 (11)	C69—H69B	0.9800
C29—H29	0.9500	C69—H69C	0.9800
C30—C31	1.367 (10)	C70—H70A	0.9800
C30—C57	1.513 (15)	C70—H70B	0.9800
C31—C32	1.401 (10)	C70—H70C	0.9800
			
C23—O1—C45	115.8 (8)	C46—C45—H45A	110.8
C47—O2—C46	113.7 (7)	O1—C45—H45B	110.8
C47—O3—C49	108.6 (9)	C46—C45—H45B	110.8
C49—O4—C48	107.1 (9)	H45A—C45—H45B	108.8
C52—O5—C54	107.0 (9)	O2—C46—C45	103.3 (8)
C54—O6—C53	109.4 (9)	O2—C46—C53	110.9 (8)
C35—O7—C58	117.1 (8)	C45—C46—C53	114.5 (8)
C60—O8—C59	114.6 (7)	O2—C46—H46	109.3
C60—O9—C62	111.3 (9)	C45—C46—H46	109.3
C61—O10—C62	107.2 (8)	C53—C46—H46	109.3
C67—O11—C65	108.5 (8)	O2—C47—O3	109.4 (8)
C67—O12—C66	107.0 (8)	O2—C47—C48	113.2 (9)
C4—N1—C1	110.7 (9)	O3—C47—C48	105.8 (9)
C4—N1—H1	124.6	O2—C47—H47	109.4
C1—N1—H1	124.6	O3—C47—H47	109.4
C9—N2—C6	101.6 (9)	C48—C47—H47	109.4
C14—N3—C11	112.4 (9)	O4—C48—C47	102.7 (10)
C14—N3—H3	123.8	O4—C48—C52	108.7 (9)
C11—N3—H3	123.8	C47—C48—C52	117.0 (10)
C16—N4—C19	106.0 (9)	O4—C48—H48	109.4
C20—C1—N1	124.0 (10)	C47—C48—H48	109.4
C20—C1—C2	129.8 (11)	C52—C48—H48	109.4
N1—C1—C2	106.2 (10)	O4—C49—O3	105.8 (9)
C3—C2—C1	109.3 (10)	O4—C49—C50	108.1 (10)
C3—C2—H2	125.4	O3—C49—C50	108.2 (11)
C1—C2—H2	125.4	O4—C49—C51	110.7 (12)
C2—C3—C4	108.5 (10)	O3—C49—C51	110.5 (10)
C2—C3—H3A	125.7	C50—C49—C51	113.2 (12)
C4—C3—H3A	125.7	C49—C50—H50A	109.5
N1—C4—C5	127.1 (10)	C49—C50—H50B	109.5
N1—C4—C3	105.3 (10)	H50A—C50—H50B	109.5
C5—C4—C3	127.5 (11)	C49—C50—H50C	109.5
C4—C5—C6	124.6 (10)	H50A—C50—H50C	109.5
C4—C5—C21	118.5 (10)	H50B—C50—H50C	109.5
C6—C5—C21	116.9 (11)	C49—C51—H51A	109.5
N2—C6—C5	124.1 (11)	C49—C51—H51B	109.5
N2—C6—C7	112.6 (9)	H51A—C51—H51B	109.5
C5—C6—C7	123.4 (11)	C49—C51—H51C	109.5
C8—C7—C6	106.5 (10)	H51A—C51—H51C	109.5
C8—C7—H7	126.8	H51B—C51—H51C	109.5
C6—C7—H7	126.8	O5—C52—C48	105.4 (9)
C7—C8—C9	105.9 (9)	O5—C52—C53	104.8 (8)
C7—C8—H8	127.1	C48—C52—C53	113.8 (9)
C9—C8—H8	127.1	O5—C52—H52	110.8
N2—C9—C10	123.9 (11)	C48—C52—H52	110.8
N2—C9—C8	113.4 (9)	C53—C52—H52	110.8
C10—C9—C8	122.7 (10)	O6—C53—C46	109.1 (9)
C11—C10—C9	126.4 (10)	O6—C53—C52	104.1 (8)
C11—C10—C27	117.6 (10)	C46—C53—C52	111.4 (8)
C9—C10—C27	116.0 (11)	O6—C53—H53	110.7
C10—C11—N3	127.4 (10)	C46—C53—H53	110.7
C10—C11—C12	127.6 (11)	C52—C53—H53	110.7
N3—C11—C12	104.9 (11)	O6—C54—O5	104.6 (10)
C13—C12—C11	108.5 (10)	O6—C54—C55	108.9 (12)
C13—C12—H12	125.8	O5—C54—C55	109.7 (13)
C11—C12—H12	125.8	O6—C54—C56	107.9 (13)
C12—C13—C14	109.1 (11)	O5—C54—C56	110.9 (12)
C12—C13—H13	125.4	C55—C54—C56	114.3 (12)
C14—C13—H13	125.4	C54—C55—H55A	109.5
C15—C14—N3	126.1 (10)	C54—C55—H55B	109.5
C15—C14—C13	128.9 (11)	H55A—C55—H55B	109.5
N3—C14—C13	105.0 (11)	C54—C55—H55C	109.5
C14—C15—C16	124.8 (11)	H55A—C55—H55C	109.5
C14—C15—C33	119.9 (9)	H55B—C55—H55C	109.5
C16—C15—C33	115.3 (11)	C54—C56—H56A	109.5
N4—C16—C15	126.7 (10)	C54—C56—H56B	109.5
N4—C16—C17	110.1 (9)	H56A—C56—H56B	109.5
C15—C16—C17	123.1 (10)	C54—C56—H56C	109.5
C18—C17—C16	105.8 (10)	H56A—C56—H56C	109.5
C18—C17—H17	127.1	H56B—C56—H56C	109.5
C16—C17—H17	127.1	C30—C57—H57A	109.5
C17—C18—C19	108.2 (10)	C30—C57—H57B	109.5
C17—C18—H18	125.9	H57A—C57—H57B	109.5
C19—C18—H18	125.9	C30—C57—H57C	109.5
C20—C19—N4	125.3 (11)	H57A—C57—H57C	109.5
C20—C19—C18	124.9 (10)	H57B—C57—H57C	109.5
N4—C19—C18	109.8 (9)	O7—C58—C59	104.4 (8)
C1—C20—C19	128.9 (10)	O7—C58—H58A	110.9
C1—C20—C39	115.6 (10)	C59—C58—H58A	110.9
C19—C20—C39	115.4 (10)	O7—C58—H58B	110.9
C22—C21—C26	116.6 (11)	C59—C58—H58B	110.9
C22—C21—C5	120.7 (11)	H58A—C58—H58B	108.9
C26—C21—C5	122.7 (9)	O8—C59—C65	111.6 (9)
C23—C22—C21	120.9 (11)	O8—C59—C58	104.0 (7)
C23—C22—H22	119.5	C65—C59—C58	114.2 (8)
C21—C22—H22	119.5	O8—C59—H59	108.9
C24—C23—O1	125.3 (10)	C65—C59—H59	108.9
C24—C23—C22	120.8 (10)	C58—C59—H59	108.9
O1—C23—C22	113.9 (10)	O8—C60—O9	109.2 (8)
C23—C24—C25	119.5 (11)	O8—C60—C61	114.3 (8)
C23—C24—H24	120.2	O9—C60—C61	103.9 (9)
C25—C24—H24	120.2	O8—C60—H60	109.7
C26—C25—C24	119.8 (11)	O9—C60—H60	109.7
C26—C25—H25	120.1	C61—C60—H60	109.7
C24—C25—H25	120.1	O10—C61—C66	106.3 (9)
C25—C26—C21	122.4 (11)	O10—C61—C60	103.5 (9)
C25—C26—H26	118.8	C66—C61—C60	116.0 (9)
C21—C26—H26	118.8	O10—C61—H61	110.2
C28—C27—C32	117.4 (11)	C66—C61—H61	110.2
C28—C27—C10	123.9 (10)	C60—C61—H61	110.2
C32—C27—C10	118.6 (9)	O9—C62—O10	104.5 (10)
C27—C28—C29	120.9 (11)	O9—C62—C63	110.0 (12)
C27—C28—H28	119.6	O10—C62—C63	108.7 (10)
C29—C28—H28	119.6	O9—C62—C64	110.5 (10)
C30—C29—C28	121.2 (11)	O10—C62—C64	109.2 (12)
C30—C29—H29	119.4	C63—C62—C64	113.5 (12)
C28—C29—H29	119.4	C62—C63—H63A	109.5
C29—C30—C31	118.7 (12)	C62—C63—H63B	109.5
C29—C30—C57	121.1 (11)	H63A—C63—H63B	109.5
C31—C30—C57	120.2 (12)	C62—C63—H63C	109.5
C30—C31—C32	120.2 (12)	H63A—C63—H63C	109.5
C30—C31—H31	119.9	H63B—C63—H63C	109.5
C32—C31—H31	119.9	C62—C64—H64A	109.5
C27—C32—C31	121.5 (11)	C62—C64—H64B	109.5
C27—C32—H32	119.2	H64A—C64—H64B	109.5
C31—C32—H32	119.2	C62—C64—H64C	109.5
C38—C33—C34	119.7 (12)	H64A—C64—H64C	109.5
C38—C33—C15	119.9 (10)	H64B—C64—H64C	109.5
C34—C33—C15	120.4 (12)	O11—C65—C59	108.0 (9)
C35—C34—C33	118.9 (12)	O11—C65—C66	103.5 (8)
C35—C34—H34	120.6	C59—C65—C66	112.7 (8)
C33—C34—H34	120.6	O11—C65—H65	110.8
C36—C35—C34	122.1 (11)	C59—C65—H65	110.8
C36—C35—O7	113.1 (10)	C66—C65—H65	110.8
C34—C35—O7	124.8 (11)	O12—C66—C61	106.4 (9)
C35—C36—C37	119.0 (12)	O12—C66—C65	104.8 (8)
C35—C36—H36	120.5	C61—C66—C65	114.4 (9)
C37—C36—H36	120.5	O12—C66—H66	110.3
C36—C37—C38	119.5 (12)	C61—C66—H66	110.3
C36—C37—H37	120.3	C65—C66—H66	110.3
C38—C37—H37	120.3	O11—C67—O12	104.1 (10)
C33—C38—C37	120.8 (11)	O11—C67—C68	109.4 (12)
C33—C38—H38	119.6	O12—C67—C68	106.9 (12)
C37—C38—H38	119.6	O11—C67—C69	110.0 (12)
C44—C39—C40	115.3 (11)	O12—C67—C69	111.5 (12)
C44—C39—C20	124.7 (10)	C68—C67—C69	114.5 (12)
C40—C39—C20	119.8 (9)	C67—C68—H68A	109.5
C39—C40—C41	123.5 (11)	C67—C68—H68B	109.5
C39—C40—H40	118.2	H68A—C68—H68B	109.5
C41—C40—H40	118.2	C67—C68—H68C	109.5
C42—C41—C40	118.8 (12)	H68A—C68—H68C	109.5
C42—C41—H41	120.6	H68B—C68—H68C	109.5
C40—C41—H41	120.6	C67—C69—H69A	109.5
C43—C42—C41	118.4 (12)	C67—C69—H69B	109.5
C43—C42—C70	122.6 (11)	H69A—C69—H69B	109.5
C41—C42—C70	118.4 (12)	C67—C69—H69C	109.5
C42—C43—C44	121.7 (11)	H69A—C69—H69C	109.5
C42—C43—H43	119.1	H69B—C69—H69C	109.5
C44—C43—H43	119.1	C42—C70—H70A	109.5
C39—C44—C43	121.6 (11)	C42—C70—H70B	109.5
C39—C44—H44	119.2	H70A—C70—H70B	109.5
C43—C44—H44	119.2	C42—C70—H70C	109.5
O1—C45—C46	105.0 (8)	H70A—C70—H70C	109.5
O1—C45—H45A	110.8	H70B—C70—H70C	109.5
			
C4—N1—C1—C20	−176.2 (10)	C58—O7—C35—C34	−4.6 (16)
C4—N1—C1—C2	1.8 (12)	C34—C35—C36—C37	3.5 (18)
C20—C1—C2—C3	175.6 (11)	O7—C35—C36—C37	−177.7 (10)
N1—C1—C2—C3	−2.3 (12)	C35—C36—C37—C38	−1.0 (18)
C1—C2—C3—C4	1.8 (13)	C34—C33—C38—C37	2.8 (18)
C1—N1—C4—C5	−176.2 (10)	C15—C33—C38—C37	−177.5 (11)
C1—N1—C4—C3	−0.8 (11)	C36—C37—C38—C33	−2.1 (18)
C2—C3—C4—N1	−0.7 (12)	C1—C20—C39—C44	−77.4 (14)
C2—C3—C4—C5	174.8 (11)	C19—C20—C39—C44	105.4 (13)
N1—C4—C5—C6	−4.9 (17)	C1—C20—C39—C40	108.5 (13)
C3—C4—C5—C6	−179.4 (11)	C19—C20—C39—C40	−68.7 (14)
N1—C4—C5—C21	174.5 (10)	C44—C39—C40—C41	5 (2)
C3—C4—C5—C21	0.1 (16)	C20—C39—C40—C41	−179.9 (13)
C9—N2—C6—C5	−179.8 (10)	C39—C40—C41—C42	1 (2)
C9—N2—C6—C7	1.8 (11)	C40—C41—C42—C43	−7 (2)
C4—C5—C6—N2	−7.9 (17)	C40—C41—C42—C70	−179.0 (13)
C21—C5—C6—N2	172.7 (10)	C41—C42—C43—C44	7 (2)
C4—C5—C6—C7	170.4 (10)	C70—C42—C43—C44	178.5 (11)
C21—C5—C6—C7	−9.0 (15)	C40—C39—C44—C43	−5.6 (17)
N2—C6—C7—C8	−2.2 (12)	C20—C39—C44—C43	180.0 (11)
C5—C6—C7—C8	179.4 (11)	C42—C43—C44—C39	−0.2 (18)
C6—C7—C8—C9	1.5 (12)	C23—O1—C45—C46	−172.6 (9)
C6—N2—C9—C10	177.7 (10)	C47—O2—C46—C45	−168.4 (8)
C6—N2—C9—C8	−0.9 (11)	C47—O2—C46—C53	68.5 (11)
C7—C8—C9—N2	−0.4 (13)	O1—C45—C46—O2	−179.6 (8)
C7—C8—C9—C10	−179.0 (10)	O1—C45—C46—C53	−58.9 (11)
N2—C9—C10—C11	12.8 (17)	C46—O2—C47—O3	84.2 (11)
C8—C9—C10—C11	−168.7 (11)	C46—O2—C47—C48	−33.6 (13)
N2—C9—C10—C27	−166.8 (9)	C49—O3—C47—O2	−117.7 (10)
C8—C9—C10—C27	11.7 (15)	C49—O3—C47—C48	4.6 (11)
C9—C10—C11—N3	−4.9 (19)	C49—O4—C48—C47	31.7 (11)
C27—C10—C11—N3	174.8 (10)	C49—O4—C48—C52	156.2 (9)
C9—C10—C11—C12	171.9 (11)	O2—C47—C48—O4	98.0 (10)
C27—C10—C11—C12	−8.5 (17)	O3—C47—C48—O4	−21.9 (10)
C14—N3—C11—C10	177.0 (11)	O2—C47—C48—C52	−20.9 (14)
C14—N3—C11—C12	−0.4 (11)	O3—C47—C48—C52	−140.8 (9)
C10—C11—C12—C13	−175.4 (11)	C48—O4—C49—O3	−29.6 (12)
N3—C11—C12—C13	2.0 (12)	C48—O4—C49—C50	−145.4 (9)
C11—C12—C13—C14	−2.7 (13)	C48—O4—C49—C51	90.1 (11)
C11—N3—C14—C15	178.2 (10)	C47—O3—C49—O4	15.0 (12)
C11—N3—C14—C13	−1.2 (11)	C47—O3—C49—C50	130.7 (9)
C12—C13—C14—C15	−176.9 (11)	C47—O3—C49—C51	−104.9 (12)
C12—C13—C14—N3	2.4 (12)	C54—O5—C52—C48	147.6 (10)
N3—C14—C15—C16	1.3 (17)	C54—O5—C52—C53	27.2 (12)
C13—C14—C15—C16	−179.4 (11)	O4—C48—C52—O5	171.9 (8)
N3—C14—C15—C33	179.1 (10)	C47—C48—C52—O5	−72.5 (12)
C13—C14—C15—C33	−1.6 (17)	O4—C48—C52—C53	−73.8 (12)
C19—N4—C16—C15	179.2 (10)	C47—C48—C52—C53	41.8 (14)
C19—N4—C16—C17	−2.2 (11)	C54—O6—C53—C46	−128.0 (11)
C14—C15—C16—N4	8.2 (17)	C54—O6—C53—C52	−9.0 (13)
C33—C15—C16—N4	−169.7 (10)	O2—C46—C53—O6	71.1 (10)
C14—C15—C16—C17	−170.4 (10)	C45—C46—C53—O6	−45.3 (12)
C33—C15—C16—C17	11.8 (15)	O2—C46—C53—C52	−43.3 (11)
N4—C16—C17—C18	2.1 (12)	C45—C46—C53—C52	−159.7 (9)
C15—C16—C17—C18	−179.1 (10)	O5—C52—C53—O6	−11.1 (11)
C16—C17—C18—C19	−1.2 (12)	C48—C52—C53—O6	−125.8 (10)
C16—N4—C19—C20	180.0 (10)	O5—C52—C53—C46	106.3 (10)
C16—N4—C19—C18	1.4 (12)	C48—C52—C53—C46	−8.4 (13)
C17—C18—C19—C20	−178.7 (10)	C53—O6—C54—O5	25.7 (14)
C17—C18—C19—N4	−0.1 (13)	C53—O6—C54—C55	142.9 (11)
N1—C1—C20—C19	4.4 (18)	C53—O6—C54—C56	−92.5 (12)
C2—C1—C20—C19	−173.1 (11)	C52—O5—C54—O6	−32.9 (14)
N1—C1—C20—C39	−172.4 (9)	C52—O5—C54—C55	−149.6 (11)
C2—C1—C20—C39	10.1 (17)	C52—O5—C54—C56	83.2 (13)
N4—C19—C20—C1	−9.0 (18)	C35—O7—C58—C59	−174.4 (9)
C18—C19—C20—C1	169.4 (11)	C60—O8—C59—C65	66.2 (11)
N4—C19—C20—C39	167.8 (9)	C60—O8—C59—C58	−170.2 (8)
C18—C19—C20—C39	−13.9 (15)	O7—C58—C59—O8	179.3 (8)
C4—C5—C21—C22	−55.8 (14)	O7—C58—C59—C65	−58.9 (12)
C6—C5—C21—C22	123.6 (11)	C59—O8—C60—O9	86.1 (10)
C4—C5—C21—C26	123.1 (11)	C59—O8—C60—C61	−29.8 (12)
C6—C5—C21—C26	−57.4 (14)	C62—O9—C60—O8	−114.6 (10)
C26—C21—C22—C23	−0.8 (16)	C62—O9—C60—C61	7.8 (11)
C5—C21—C22—C23	178.2 (10)	C62—O10—C61—C66	154.0 (9)
C45—O1—C23—C24	−0.3 (17)	C62—O10—C61—C60	31.4 (10)
C45—O1—C23—C22	179.6 (9)	O8—C60—C61—O10	95.2 (10)
C21—C22—C23—C24	1.9 (18)	O9—C60—C61—O10	−23.7 (10)
C21—C22—C23—O1	−177.9 (10)	O8—C60—C61—C66	−20.8 (14)
O1—C23—C24—C25	177.6 (10)	O9—C60—C61—C66	−139.8 (9)
C22—C23—C24—C25	−2.2 (18)	C60—O9—C62—O10	11.0 (12)
C23—C24—C25—C26	1.5 (17)	C60—O9—C62—C63	127.6 (9)
C24—C25—C26—C21	−0.4 (18)	C60—O9—C62—C64	−106.4 (12)
C22—C21—C26—C25	0.1 (17)	C61—O10—C62—O9	−26.9 (12)
C5—C21—C26—C25	−178.9 (11)	C61—O10—C62—C63	−144.3 (10)
C11—C10—C27—C28	76.5 (15)	C61—O10—C62—C64	91.4 (11)
C9—C10—C27—C28	−103.8 (13)	C67—O11—C65—C59	−138.0 (10)
C11—C10—C27—C32	−105.2 (14)	C67—O11—C65—C66	−18.4 (12)
C9—C10—C27—C32	74.4 (14)	O8—C59—C65—O11	67.0 (10)
C32—C27—C28—C29	2.6 (17)	C58—C59—C65—O11	−50.6 (12)
C10—C27—C28—C29	−179.1 (10)	O8—C59—C65—C66	−46.6 (12)
C27—C28—C29—C30	−2.1 (18)	C58—C59—C65—C66	−164.2 (9)
C28—C29—C30—C31	2.1 (19)	C67—O12—C66—C61	144.7 (9)
C28—C29—C30—C57	179.0 (11)	C67—O12—C66—C65	23.2 (11)
C29—C30—C31—C32	−3 (2)	O10—C61—C66—O12	166.9 (8)
C57—C30—C31—C32	−179.5 (13)	C60—C61—C66—O12	−78.7 (11)
C28—C27—C32—C31	−3 (2)	O10—C61—C66—C65	−77.9 (11)
C10—C27—C32—C31	178.5 (13)	C60—C61—C66—C65	36.5 (14)
C30—C31—C32—C27	3 (2)	O11—C65—C66—O12	−3.2 (10)
C14—C15—C33—C38	−124.1 (12)	C59—C65—C66—O12	113.3 (10)
C16—C15—C33—C38	53.9 (14)	O11—C65—C66—C61	−119.3 (9)
C14—C15—C33—C34	55.6 (15)	C59—C65—C66—C61	−2.8 (14)
C16—C15—C33—C34	−126.4 (11)	C65—O11—C67—O12	33.1 (12)
C38—C33—C34—C35	−0.5 (17)	C65—O11—C67—C68	147.0 (11)
C15—C33—C34—C35	179.8 (10)	C65—O11—C67—C69	−86.4 (12)
C33—C34—C35—C36	−2.7 (18)	C66—O12—C67—O11	−34.9 (12)
C33—C34—C35—O7	178.6 (11)	C66—O12—C67—C68	−150.6 (10)
C58—O7—C35—C36	176.6 (10)	C66—O12—C67—C69	83.6 (12)

### meso-5,15-Bis{4-[{4,4,11,11-tetramethyl-3,5,7,10,12-pentaoxatricyclo[7.3.0.02,6]dodecan-8-yl)methoxy]phenyl}-10,20-bis(4-methylphenyl)porphyrin Hydrogen-bond geometry (Å, º)

π1, π2, π3 and π4 are the centroids of the [please define] rings, respectively.

**Table:**

*D*—H···*A*	*D*—H	H···*A*	*D*···*A*	*D*—H···*A*
C45—H45*B*···O4^i^	0.99	2.55	3.18 (1)	121
C24—H24···O4^i^	0.95	2.60	3.54 (1)	169
C48—H48···π1^i^	1.00	3.18	4.120	165
C53—H53···O3^ii^	1.00	2.70	3.67 (1)	165
C40—H40···π3^iii^	0.95	2.89	3.828	171
C32—H32···π4^iv^	0.95	2.83	3.782	176
C58—H58*B*···O10^v^	0.99	2.70	3.19 (1)	110
C61—H61···π2^vi^	1.00	3.20	4.117	153
C36—H36···O9^vi^	0.95	2.70	3.56 (1)	152

Symmetry codes: (i) −*x*+1, *y*−1/2, −*z*; (ii) −*x*+1, *y*+1/2, −*z*; (iii) *x*, *y*+1, *z*; (iv) *x*, *y*−1, *z*; (v) −*x*+3, *y*−1/2, −*z*+1; (vi) −*x*+3, *y*+1/2, −*z*+1.
